# Mosquito E-20-Monooxygenase Gene Knockout Increases Dengue Virus Replication in *Aedes aegypti* Cells

**DOI:** 10.3390/v16040525

**Published:** 2024-03-28

**Authors:** Bo Li, Di Wang, Xiaoxue Xie, Xiaoli Chen, Guorui Liang, Dan Xing, Teng Zhao, Jiahong Wu, Xinyu Zhou, Chunxiao Li

**Affiliations:** 1 School of Public Health, The Key Laboratory of Environmental Pollution Monitoring and Disease Control, Ministry of Education, Guizhou Medical University, Guiyang 550025, China; 2 State Key Laboratory of Pathogen and Biosecurity, Beijing Institute of Microbiology and Epidemiology, Beijing 100071, China; 3 The Key and Characteristic Laboratory of Modern Pathogen Biology, College of Basic Medicine, Guizhou Medical University, Guiyang 550025, China

**Keywords:** E-20-monooxygenase, CRISPR/Cas9, DENV2, overexpression

## Abstract

E-20-monooxygenase (E20MO) is an enzymatic product of the shade (shd) locus (cytochrome p450, E20MO). Initially discovered in Drosophila, E20MO facilitates the conversion of ecdysone (E) into 20-hydroxyecdysone (20E) and is crucial for oogenesis. Prior research has implicated 20E in growth, development, and insecticide resistance. However, little attention has been given to the association between the E20MO gene and DENV2 infection. The transcriptome of Ae. aegypti cells (Aag2 cells) infected with DENV2 revealed the presence of the E20MO gene. The subsequent quantification of E20MO gene expression levels in Aag2 cells post-DENV infection was carried out. A CRISPR/Cas9 system was utilized to create an E20MO gene knockout cell line (KO), which was then subjected to DENV infection. Analyses of DENV2 copies in KO and wild-type (WT) cells were conducted at different days post-infection (dpi). Plasmids containing E20MO were constructed and transfected into KO cells, with pre- and post-transfection viral copy comparisons. Gene expression levels of E20MO increased after DENV infection. Subsequently, a successful generation of an E20MO gene knockout cell line and the verification of code-shifting mutations at both DNA and RNA levels were achieved. Furthermore, significantly elevated DENV2 RNA copies were observed in the mid-infection phase for the KO cell line. Viral RNA copies were lower in cells transfected with plasmids containing E20MO, compared to KO cells. Through knockout and plasmid complementation experiments in Aag2 cells, the role of E20MO in controlling DENV2 replication was demonstrated. These findings contribute to our understanding of the intricate biological interactions between mosquitoes and arboviruses.

## 1. Introduction

*Aedes aegypti* (*Ae. aegypti*) is an endemic mosquito vector distributed worldwide, particularly in tropical and subtropical environments [1]. This distribution enables the primary transmission of multiple viruses to humans, including the dengue virus (DENV), Chikungunya virus (CHIKV), and Zika virus (ZIKV) [2,3]. Based on cartographic approaches, more than 100 dengue-endemic countries have been identified, with 390 million people infected annually [4]. DENV, which consists of four distinct serotypes, can cause a range of disease symptoms, from a self-limited febrile illness known as dengue fever (DF) to severe conditions such as dengue hemorrhagic fever (DHF) and dengue shock syndrome (DSS) [5,6]. The binding and entry of DENV into host cells is mediated by multiple receptors, followed by the internalization of virus particles and the release of viral RNA into the cytoplasm through endosomal acidification. Subsequently, the positive-sense RNA genome is transcribed and translated into a large, single polyprotein, which is then cleaved by host and viral proteases. The structure of DENV consists of three structural proteins, the capsid protein C, the membrane protein precursor M (prM) (which matures into the membrane protein M after virus particle maturation), and the envelope protein E, as well as seven nonstructural proteins (NS1, NS2a, NS2b, NS3, NS4a, NS4b, and NS5) [7].

E-20-monooxygenase (E20MO) is the product of the gene *shade* (*shd*) locus (cytochrome p450, E20MO) [8]. As first reported in Drosophila, E20MO converts ecdysone (E) into 20-hydroxyecdysone (20E) [8,9], and it is required for oogenesis [8]. Additionally, conversion by E20MO has also been described in other species, including the cotton bollworm [10], blackback land crab [11], and small brown planthopper *Laodelphax striatellus* [12], which influences development through 20E. In *Anopheles gambiae*, anti-Plasmodium immunity is promoted via a 20E agonist, which enhances innate immune responses related to bacterial and malarial parasite survival [13,14]. E20MO also plays a role in regulating the sexually dimorphic metamorphosis of *Ericerus pela* [15]. Acetamiprid and phoxim resistances in melon aphids and silkworms [16,17] are also attributed to the effect of E20MO. In *Aedes aegypti*, the expression of E20MO is mainly observed in the ovaries, gut, and abdominal wall [18]. The trade-off between immunity and reproduction in *Aedes aegypti* may be governed by 20E [19]. In summary, most studies on 20E, rather than E20MO, are associated with development, insecticide resistance, and immunity against bacteria or malaria parasites. However, there are few studies on the involvement of E20MO and the shade locus in arbovirus infection or innate immunity.

The E20MO gene was screened according to the transcriptome of Aag2 cells infected with DENV2. The results showed that the E20MO gene was upregulated after DENV2 infection in Aag2 cells [20]. E20MO knockout (KO) Aag2 cells were constructed to investigate the effect and mechanism of the E20MO gene in response to DENV2 in Aag2 cells. To compare and evaluate the viral load (VL) between the KO and wild-type (WT) cells, extracellular viral RNA from particles was quantified by RT-qPCR following the replication and assembly of viral particles. The results showed that E20MO KO cells resulted in an increase in extracellular DENV2 copies, which suggests that the E20MO gene may be closely related to dengue virus replication in Aag2 cells. The results from this study improve our understanding of the complex biological interactions between mosquitoes and arboviruses.

## 2. Materials and Methods

### 2.1. Virus and Cells

The DENV2 Guangdong strain was provided by the Guangdong Provincial Centers for Disease Control and Prevention in China [21]. *Ae. aegypti* Aag2 cells were cultured in Schneider’s Drosophila medium (SDM, Gibco, Penrose, NZ, AUK) supplemented with 10% fetal bovine serum (FBS, Gibco, Penrose, NZ, AUK) at 28 °C with 5% CO_2_. To amplify DENV2, C6/36 cells were cultured in RPMI medium 1640 (RPMI 1640, Gibco, Penrose, NZ, AUK) containing 10% FBS at 28 °C and 5% CO_2_. BHK-21 cells were used for plaque formation to determine DENV2 titers.

### 2.2. Design and Synthesis of sgRNA Targets

To investigate the role of the E20MO gene in DENV2 replication in Aag2 cells, we employed the CRISPR/Cas9 system to create an E20MO knockout (KO) cell line. In vitro, a ribonucleoprotein (RNP) complex was formed by chemically synthesizing sgRNA and Cas9 proteins, thus enhancing sgRNA stability while reducing off-target effects [22,23]. The DNA sequence of the *Aedes aegypti* E20MO gene (gene no. 110677901) was obtained from the NCBI database (https://www.ncbi.nlm.nih.gov/ (accessed on 18 June 2021)) and accessed through the Benching website (https://benching.com/ (accessed on 18 June 2021)). The target sequence for sgRNA1 was 5′-AACGACGATACACGTCCCACCGG-3′, and that for sgRNA2 was 5′-TTGCGCGTCAAGCTAACCTCAGG-3′. The sgRNA sequences were synthesized by GenScript Biotech.

### 2.3. Construction of E20MO KO Aag2 Cells

Chemical synthetic sgRNA and Cas9 proteins (632678, TAKARA, Osaka, Japan) were delivered into cells through electrical transfer. Ribonucleoprotein (RNP) complexes were formed by combining sgRNA and Cas9 proteins in vitro. RNP complex 1 (20 µL) was prepared by combining 8 µL of Guide-it™ Recombinant Cas9 (10 µg/µL) with 12 µL of sgRNA1 solution (0.1 nM/µL in water). Similarly, RNP complex 2 (20 µL) was prepared by mixing 8 µL of Guide-it™ Recombinant Cas9 (10 µg/µL) with 12 µL of sgRNA2 solution (0.1 nM/µL in water). After individual mixing for 10 min, the two RNP complexes were combined and further mixed for 5–10 min to obtain a final RNP complex of 40 µL. The wild-type Aag2 cells were washed once with PBS, and the cell pellet was collected for electroporation, with a cell number range of 5 × 10^4^ to 1 × 10^6^. Preparation of the electroporation buffer (total volume 40 µL) involved mixing 32.8 µL of P3 Primary Cell Nucleofector^®^ Solution with 7.2 µL. Both the P3 Primary Cell Nucleofector^®^ Solution and Supplement 1 were obtained from the Primary Cell 4D-Nucleofector™ X Kit S (V4XP-3032, Lonza, Tokyo, Japan). A mixture of two sgRNAs and Cas9 proteins was introduced into wild-type Aag2 cells using an electroporation system, and the cells were cultured for 6 days for the primary screening of mutants. The collected Aag2 cells were then resuspended in a mixture of RNP complexes and an electrotransfer buffer (Lonza) before being transferred to an electrotransfer cup. The electrotransfer apparatus (Lonza) was run using the K562 program for five minutes. After electrotransformation, the cells were resuspended in SDM medium containing 10% fetal bovine serum (FBS) and incubated at 28 °C. Subsequently, the cells were incubated using the K562 program for five minutes. When the cells reached 80–90% confluency in the culture dish, they were visually inspected under a microscope, and individual cells with a well-defined morphology, intact and clear edges, moderate size, and homogeneous transparency were selected to transfer into a 96-well plate. Subsequently, we sequentially transferred the cells from the 96-well plate to the 12-well plate and 6-well plate for amplification culture, aiming to obtain the final gene knockout cells.

### 2.4. E20MO KO Cell Detection at the DNA Level

After the cells reached a certain confluence, DNA was extracted from the cells (Insect DNA Extraction Kit from Shanghai Enlighten Biotech, Shanghai, China). The extracted DNA was then amplified through polymerase chain reaction (PCR) using the following specific primer sequences: forward primer (5′-GTGGTCGCCTTCTGCTAT-3′) and reverse primer (5′-TTGCTTGTCTCCGCTCAT-3′). Subsequently, the amplified products were analyzed using agarose gel electrophoresis and subjected to sequencing. For the PCR system, the following components were included: 2 × EasyTaq^®^ PCR SuperMix from TransGen Biotech (Beijing, China) at 12.5 μL, 10 μM forward primer at 0.5 μL, 10 μM reverse primer at 0.5 μL, DNA template at 2 μL, and nuclease-free water to bring the total reaction volume to 25 μL. PCR was carried out as follows: initial denaturation at 94 °C for 10 min, followed by 35 cycles of denaturation at 94 °C for 30 s, annealing at 60 °C for 30 s, extension at 72 °C for 1 min 15 s, and a final extension at 72 °C for 10 min. PCR products were analyzed by agarose gel electrophoresis, and selected PCR products were sent to the Tianyi Huiyuan Company (Beijing, China) for sequencing.

### 2.5. qRT-PCR Analysis of E20MO Expression in Ae. aegypti and Aag2 Cells

RNA was extracted from KO Aag2 cells and WT Aag2 cells (including DENV2-infected and noninfected cells) with TRIzol, and TransScript^®^ Green One-Step qRT-PCR SuperMix (TransGen Biotech, Beijing, China) was used for quantitative reverse transcription PCR (qRT-PCR) with RNA as the template. The forward primer 5′-TGCCATGCAGATGAATCAAGC-3′ and reverse primer (5′-GGTTCACGATTGGCACATGA-3′) were used as the qRT-PCR primer sequences. The reaction system consisted of 2 × PerfectStartTM Green One-Step qPCR SuperMix (10 μL), TransScript^®^ Green One-Step RT/RI Enzyme Mix (0.4 μL), forward primer (10 μM) (0.4 μL), reverse primer (10 μM) (0.4 μL), passive reference dye (50×) (0.4 μL), RNA template (2 μL), and nucleic acid-free water (to 20 μL). The reaction procedure was as follows: 45 °C for 5 min and 40 cycles of 94 °C for 30 s, 94 °C for 5 s, and 60 °C for 30 s.

### 2.6. Viral Infection of KO and WT Aag2 Cells

Two milliliters of KO and WT Aag2 cell suspension (5% FBS) (1 × 10^6^ cells/mL) were seeded into 12-well plates. After 30 min of incubation, DENV2 was diluted to the appropriate ratio in SDM medium containing 5% FBS to obtain a multiplicity of infection (MOI) of 0.1. Next, 100 μL of the diluted virus was added to each cell plate, and the cells were cultured in an incubator at 28 °C with 5% CO_2_. One milliliter of WT and DWT cell groups (1 × 10^5^ cells/mL) were seeded instead, and MOI was adjusted to 0.01. After infection, first, 10 μL cell suspension was mixed with trypan blue solution at a ratio of 1:1; then, the 10 μL mixture was used to determine the cell concentration. After the centrifugation of the cell suspension, 100 μL of the cell supernatant was taken daily and stored at −80 °C until the 7th day post-infection (dpi). The experiment was designed in two groups, with four biological replicates: the KO group (KO Aag2 cells + DENV2) and the WT group (WT Aag2 cells + DENV2).

### 2.7. Plasmid for E20MO Synthesis and Extraction

The PSL1180polyUBdsRED plasmid, which can express red fluorescent protein, was designed by Snapgene software 6.0.2 using dual-enzyme digestion [24] to avoid generating scrambled and reverse ligation. Enhanced green fluorescent protein (EGFP) and green fluorescent protein-puromycin sites were added to the original vector, and the plasmid was constructed by replacing the EGFP fragment with the CDS fragment of the target gene. After the carrier was designed, it was synthesized by Bio-engineering (Shanghai) Co. Ltd. (Shanghai, China). Ten microliters of plasmid containing each gene was added to 100 mL of liquid LB medium, followed by incubation at a constant temperature (37 °C 200 R/min) for 12 h. Then, 30 mL of plasmid was centrifuged at 8000 R/min for 3 min, and the supernatant (medium) was removed; this enrichment process was repeated four times. The plasmids were extracted by an endotoxin-free plasmid extraction kit and then stored at −20 °C (Lipopolysaccharide-free Plasmid Extraction Kit DP117, Tiangen biochemical technology., Beijing, Co., Ltd., Beijing, China).

### 2.8. Plasmid Containing E20MO/EGFP Replenishment and Viral Infection of Cells

One milliliter of Aag2 cell suspension (5% FBS) (1 × 10^6^ cells/mL) was seeded into 12-well plates. After 2 days of incubation, plasmids encoding E20MO and EGFP were transfected into KO cells using FuGENE Transfection Reagent (FuGENE^®^, Promega, Madison, WI, USA). After 24 h and after observing the cells under a fluorescence microscope, some cells were used to measure the relative gene expression of E20MO. The rest of the cells were utilized for viral cell infection. DENV2 was diluted to the appropriate concentration in SDM medium containing 5% FBS to obtain an MOI of 0.1. Subsequently, 100 μL of the diluted virus was added to each cell plate, and the cells were incubated in an incubator at 28 °C with 5% CO_2_. One hundred microliters of the cell supernatant were collected on the 2nd, 4th, and 6th dpi and stored at −80 °C. The experiment was designed with four groups, each having three biological replicates: the KOE group (KO Aag2 cells + EGFP plasmid + DENV2), KOC group (KO Aag2 cells + E20MO plasmid + DENV2), KO group (KO Aag2 cells + DENV2), and WT group (WT Aag2 cells + DENV2).

### 2.9. Analysis of Extracellular Viral Copies in the Cell Supernatant

DENV2 RNA was detected using a DENV2 non-nucleic acid detection kit from Beijing Merab Medical Technology. The reaction mixture consisted of 2× DENV2 amplification solution (10 μL), DENV2 primer and probe mixture (1 μL), reverse transcription PCR (RT-PCR) enzyme mixture (1 μL), cell supernatant (4 μL), and nuclease-free water to bring the reaction volume to 20 μL. The reaction conditions were as follows: 40 cycles of DENV2 amplification with the following temperature profile: 50 °C for 10 min and 95 °C for 10 min, followed by denaturation at 95 °C for 15 s and annealing/extension at 60 °C for 30 s. The primers used were as follows: forward primer (5′-AATTAGAGCAGATCTGATGAA-3′), reverse primer (5′-AGCATTCCAAGTGAGAATCTCTTTGT-3′), and DENV2 primer and probe (5′-AGCATTCCAAGTGAGAATCTCTTTGTCA-3′).

### 2.10. Statistical Methods

All data are presented as the means ± standard deviations (SDs). The data were processed using Excel 2019 for analysis, and figures were created using GraphPad Prism 8.0 software. Statistical analysis was conducted using SPSS software 23.0.0.0. For data with a normal distribution and homogeneity of variance, unpaired t-tests were employed. In data for which the homogeneity of variance assumption was violated, the unpaired t-test with Welch’s correction was applied. For data with a nonnormal distribution, Mann–Whitney and Kolmogorov–Smirnov tests were utilized. For comparing multiple samples that followed a normal distribution, one-way ANOVA followed by Dunnett’s method was employed. In contrast, when comparing multiple samples with a nonnormal distribution, the Kruskal–Wallis test was used, followed by Dunnett’s post hoc analysis.

## 3. Results

### 3.1. The E20MO Gene Was Upregulated after DENV2 Infection

**Figure 1:**
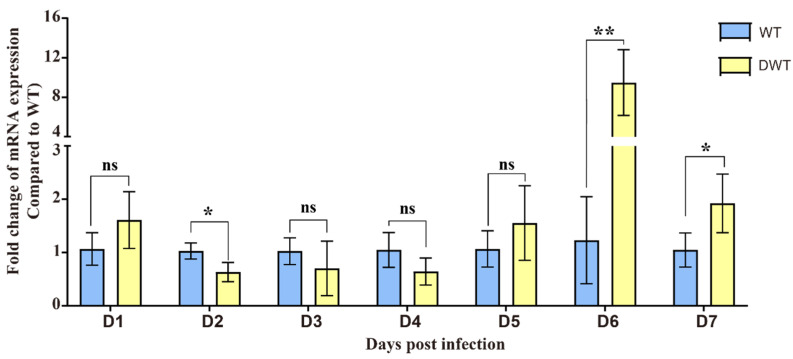
Quantitative reverse transcription polymerase chain reaction (qRT-PCR) was performed to assess the expression level of E20MO mRNA in two types of Aag2 cell lines from the 1st to 7th day after DENV2 infection. Abbreviations: WT, wild-type Aag2 cells; DWT, DENV2-infected wild-type Aag2 cells. The expression of E20MO mRNA in WT Aag2 cells was used as the control, with ribosomal protein S6 serving as the reference gene. Relative expression levels were calculated using the 2^−ΔΔCt^ method. The data are presented as means ± standard deviations (SDs), with * indicating statistical significance at *p* < 0.05; with ** indicating statistical significance at *p* < 0.01; and ns indicating no statistical significance.

The Aag2 cell line is an embryonic cell line of *Aedes aegypti*, and dengue virus can replicate continuously in this cell line. To analyze the expression of the E20MO gene in Aag2 cells infected with DENV2, we used qRT-PCR to assess the relative fold change (FC) of the E20MO gene expression in WT Aag2 cells from 1 to 7 dpi. The data are from independent infection time courses. Our results showed that at 6 and 7 dpi, the E20MO gene was upregulated in WT Aag2 cells infected with DENV2 (DWT) (Figure 1).

### 3.2. Deletion of 900 bp of the E20MO Gene in Aag2 KO Cells

**Figure 2:**
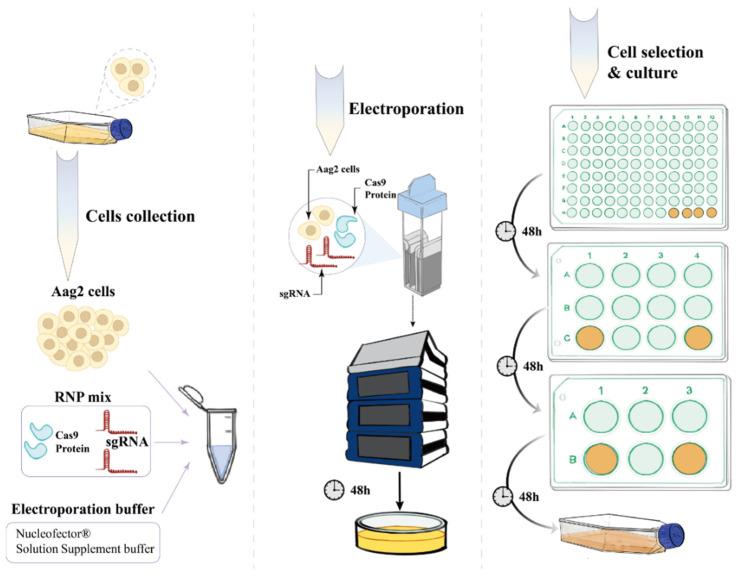
Construction of E20MO-knockout (KO) Aag2 cells.

We first collected wild-type Aag2 cells. Then, the RNP mix, which included Cas9 protein and sgRNA, and the electroporation buffer were introduced into the cells through electroporation. Next, we selected the KO cell line by PCR and cultured it for subsequent experiments (Figure 2).

**Figure 3:**
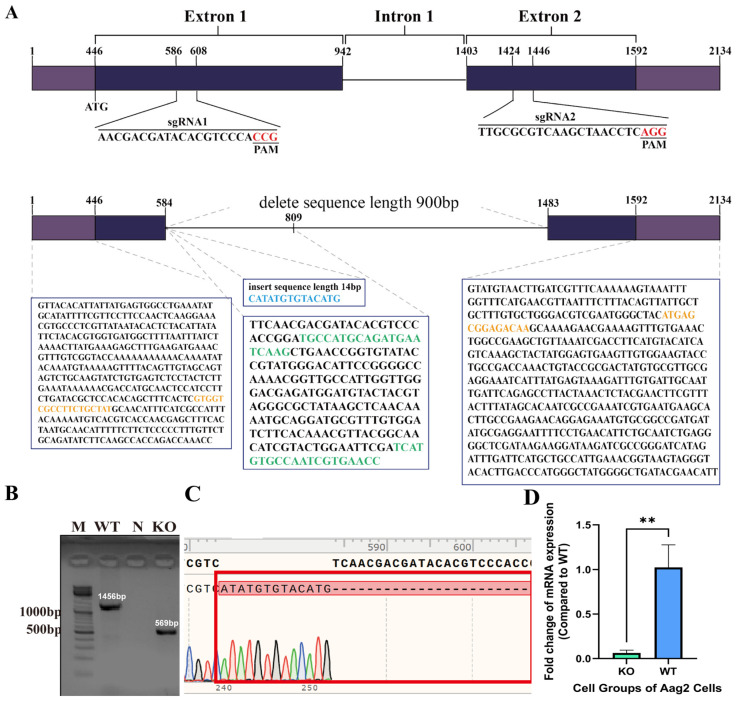
Deletion of the E20MO gene in knockout (KO) cells. (**A**) E20MO KO approach. We employed a single-guide RNA (sgRNA) strategy targeting the initial two exons, with the sgRNA sequences and target sites delineated in the illustration. In the KO cells, 900 bp within the E20MO coding region was deleted, leading to a frameshift mutation. The highlighted yellow sequence denotes the primers used for PCR analysis. The green-highlighted portion denotes the primer utilized for RT-qPCR to assess mRNA expression relative to the WT cells. The blue sequence represents the inserted sequence in the KO cells. (**B**) The agarose gel of the PCR-amplified bands. (**C**) Comparative analysis of peak profiles between two KO WT cell lines. The regions with alterations are enclosed within the red box, and the peak profile demonstrates the clonality of the selected cell line. (**D**) Quantification of E20MO mRNA expression in E20MO knockout (KO) Aag2 cell lines. The level of E20MO mRNA expression in WT Aag2 cells served as the control, with ribosomal protein S6 used as the reference gene. Relative expression was determined using the 2^−ΔΔCt^ method. The data are presented as means ± standard deviations (SDs), ** *p* < 0.01. Abbreviations: M, marker; N, negative control; WT, wild-type; KO, knockout.

In the CRISPR/Cas9 gene-editing strategy, two pairs of sgRNAs were designed at positions 586–608 bp in exon 1 and 1424–1446 bp in exon 2. As seen in Figure 3A, in the KO cells, the gene sequence from 584–1483 bp (900 bp) was deleted, and 14 bases were inserted. Sequencing peaks for the KO sequence and the WT sequence are depicted in Figure 3B; the altered sequence sites are highlighted in the red box, and the peak profile confirms that the KO cell lines consist of monoclonal cells.

### 3.3. Lack of Expression of the E20MO Gene in KO Cells

To further verify the 900 bp deletion at the mRNA level, qRT-PCR was utilized to measure the expression levels of E20MO mRNA in KO cells. KO cells and WT cells were cultured separately at the same time, and RNA was extracted from the same number of cells to analyze E20MO mRNA. The primers were designed to target the sites of deleted sequences in the KO cell lines (Figure 3A). For each experiment, there were three replicates each for the KO and WT groups. E20MO mRNA was detected in the WT Aag2 cells but was barely detectable in KO Aag2 cells. The significant decrease in the relative expression of the E20MO gene in KO Aag2 cells is illustrated in Figure 3C. In conclusion, E20MO was successfully knocked out at both the DNA and mRNA levels in the KO cell lines.

### 3.4. Viral Replication Is Elevated in KO Cells

**Figure 4:**
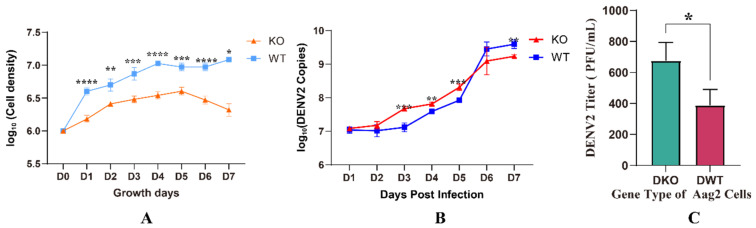
Quantification of extracellular dengue virus 2 (DENV2) RNA in supernatants of knockout (KO) cells. (**A**) The log_10_ of live cell densities of knockout (KO) cells and wild-type (WT) cells. (**B**) The number of extracellular DENV2 RNA copies was measured at 1, 2, 3, 4, 5, 6, and 7 days post-DENV2 infection. (**C**) The titers of the virus of Aag2 cells on the 4th day post-DENV2 infection. The data are presented as means ± standard deviations (SDs); * *p* < 0.05, ** *p* < 0.01, *** *p* < 0.001, and **** *p* < 0.0001. Abbreviations: WT, wild-type; DENV2, dengue virus 2; KO, knockout.

To analyze the effect of the E20MO gene on virus replication, extracellular viral RNA copies were measured at 1, 2, 3, 4, 5, 6, and 7 dpi with DENV2 in KO and WT Aag2 cells. We initially quantified the live cell density for the two groups, as presented in Figure 4A, where a significant difference in cell density between the knockout (KO) and wild-type (WT) cells is evident. Additionally, the results of viral RNA copies demonstrate that extracellular viral RNA copies exhibited an increasing trend as the duration of infection increased. Although the live cell density of KO cells was lower than that of WT cells at 3, 4, and 5 dpi, the virus replication was higher in KO cells than in WT cells on these days (a significant increase in KO cells is indicated in Figure 4B).

In order to verify the viral activity and titer in the supernatants of Aag2 cells infected with DENV2, the supernatants of KO and WT Aag2 cells on the 4th day after infection were used for the virus titration experiment. The result showed that the virus titer of KO Aag2 cells was 675 ± 119.0 PFU/mL, and the one of WT Aag2 cells was 387.5 ± 103.1 PFU/mL (*p* < 0.05).

### 3.5. The Replication of DENV2 Was Higher in Infected KO Cells Than in KO Cells Transfected with Plasmids Containing the E20MO Gene

**Figure 5:**
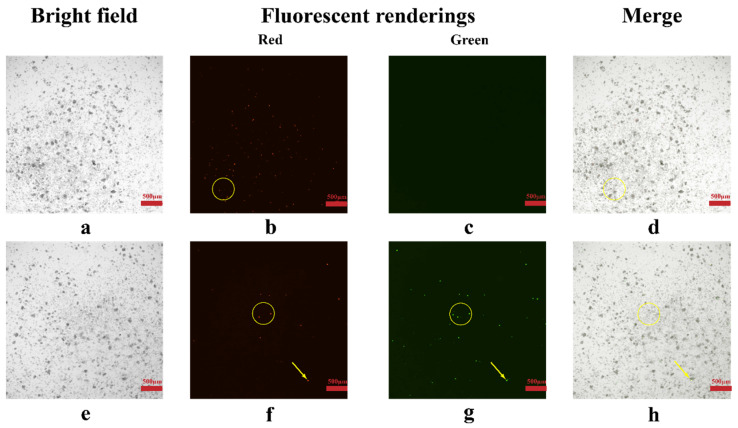
Observation of plasmid transfer by fluorescence microscopy. (**a**–**d**) are KO cells transformed with plasmids containing the E20MO gene. (**e**–**h**) are KO cells transformed with plasmids containing EGFP. The yellow circles and arrows represent the cells successfully transfected with the plasmids observed under the microscope.

To further illustrate the function of the E20MO gene in virus replication in Aag2 cells, plasmids containing the E20MO gene and plasmids containing EGFP were constructed and then transferred into KO cell lines. Transfer of the plasmids was preliminarily confirmed by fluorescence microscopy (Figure 5). Red and green fluorescence could be observed in the KO + EGFP group, and only red fluorescence could be observed in the Ko + E20MO group.

**Figure 6:**
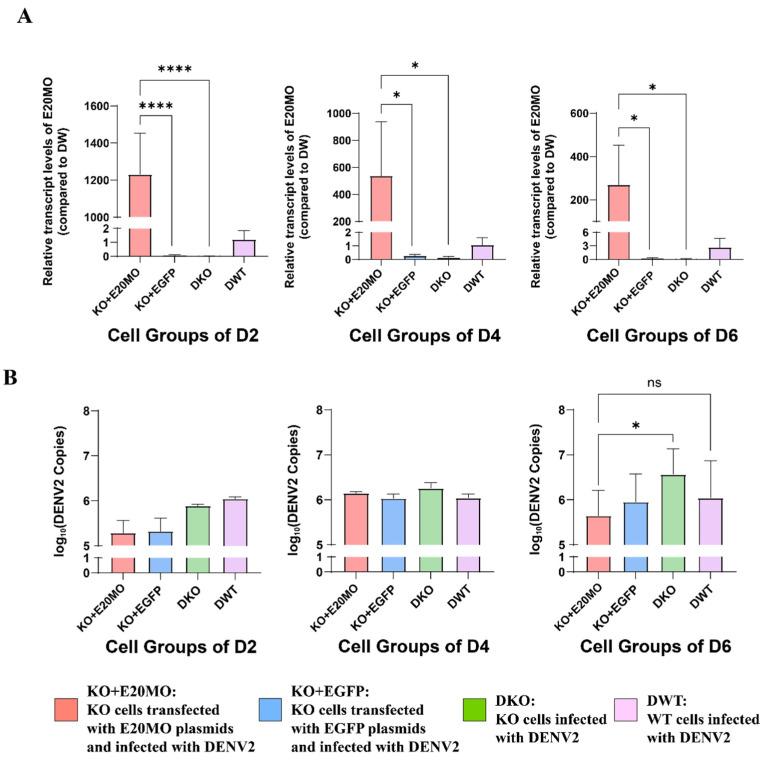
Quantification of E20MO mRNA expression and DENV2 viral RNA expression in four cell groups in the E20MO replenishment experiment. (**A**) Extracellular DENV2 RNA copies at 2, 4, and 6 dpi. (**B**) E20MO mRNA expression were measured at 2, 4, and 6 dpi. The level of E20MO mRNA expression in wild-type Aag2 cells infected with DENV2 (DWT) served as the control, with ribosomal protein S6 used as the reference gene. Relative expression was calculated using the 2^−ΔΔCt^ method. The data are presented as means ± standard deviations (SDs). Ns: no significance, * *p* < 0.05, and **** *p* < 0.0001.

To further determine the success of plasmid transfer, the relative expression of E20MO mRNA was assessed at 2, 4, and 6 dpi. The relative expression of E20MO mRNA in KO cell lines transformed with plasmids containing the E20MO gene was significantly increased at 2, 4, and 6 dpi (Figure 6A). The expression level of the E20MO gene was higher in the KO + E20MO group than in the KO + EGFP group and the KO group infected with DENV2 (DKO). In summary, the plasmids containing the E20MO gene were successfully transmitted into the KO cell lines.

Extracellular viral RNA at 2, 4, and 6 dpi were measured by RT-qPCR. There was significantly less viral RNA in the KO + E20MO group than in the DKO group, and there was no significant difference in viral RNA amount between the KO + E20MO group and the group of WT cells infected with DENV2 (DWT) at 6 dpi. Due to the possible lack of 100% transfection efficiency, differences in DENV2 copy numbers were not observed between the KO + E20MO and DKO groups on the 2nd and 4th day post-infection, while a statistically significant difference was observed only on the 6th day post-infection.

## 4. Discussion

Dengue fever is considered the most prevalent and fastest spreading viral disease in the population and is mediated by mosquitoes [25]. At present, it is a major public health issue that endangers human life and health and hinders social and economic development. *Aedes aegypti*, as the main vector of dengue virus (DENV) transmission, has promoted the outbreak of dengue fever in tropical and subtropical regions [4]. Therefore, controlling the transmission vector of the dengue virus and cutting off the transmission route are the main measures to prevent and control the spread of dengue fever.

The main function of E20MO is to catalyze the generation of 20E. Most studies on the E20MO gene have been conducted in organisms such as cotton bollworms, black-backed land crabs, gray planthoppers, melon aphids, and silkworms [10,11,12,16,17]. Discussions have focused on 20E, which has been shown to have functions pertaining to growth, development, and insecticide resistance. However, there is relatively little research on the relationship between the E20MO gene and DENV2 infection in mosquitoes. Notably, in a previous study, we found the E20MO gene through screening transcriptome data of *Aedes aegypti* Aag2 cells infected with DENV2 [20]. This study focused on exploring the effect of the E20MO gene on DENV2 replication in Aag2 cells.

After exposure to exogenous DENV2, there was no significant change in the expression of the E20MO gene in the early stages, but at 6 dpi, the E20MO gene was significantly upregulated. This suggests that the expression of the E20MO gene may be related to DENV2 infection. Even though the relative expression of the E20MO gene did not differ significantly from 2 dpi to 5 dpi, it may have played a role in DENV2 infection, and differences in mRNA could be detected on day 6. After successfully constructing an E20MO KO cell line, a significant difference in cell density was observed between KO and WT cells, which may be attributed to the compromised cellular health in Aag2 cells following the knockout of the E20MO gene. Following the infection of the cell line with the virus, there was an ascending trend in the DENV2 viral load, with significant differences compared to the DENV2 viral load in the control group. Although the viral load in WT cells was higher than that in KO cells at 6 and 7 dpi, this result may be due to the poor growth and development of KO cells and the damage caused by the virus, resulting in a significantly lower cell density than that of WT cells, thus leading to a decrease in viral load. This result indicates that after the loss of the gene, the decrease in E20MO in Aag2 cells leads to an increase in the DENV2 viral load, suggesting that the E20MO gene may affect DENV2 replication in Aag2 cells. To further validate this function, we transferred plasmids containing the E20MO gene into Aag2 cells, which was confirmed by a fluorescence microscope and qRT-PCR, and infected the cells with DENV2. For cells that were successfully transformed with the plasmid, the viral load was significantly higher in KO cells than in cells transformed with the E20MO gene plasmids. This may be due to the overexpression of the E20MO gene in the Aag2 cells, which hindered the replication of DENV2 within the cell. This further suggests that the E20MO gene may hinder DENV2 replication.

Previous investigations conducted with Drosophila [26,27,28] have demonstrated that 20E signaling plays a role in regulating the synthesis of antimicrobial proteins (AMPs) [26,27]. This regulation is achieved through both PGRP-LC-dependent and PGRP-LC-independent mechanisms, mediated by the ecdysone receptor and ultraspiracle heterodimer [27]. Additional evidence from Drosophila suggests that 20E also enhances the activation of hemocytes, resulting in increased mobility, responsiveness to injury, and phagocytic activity [28,29]. This indicates that Aag2 cells may regulate the immune mechanism through the E20MO gene, thereby inhibiting the replication and spread of the dengue virus within cells.

Furthermore, Rebekah A. Reynolds et al. found that the hormone 20E plays a role in preparing mosquito innate immunity against bacteria and malaria parasites, partly due to the activation of cellular immune pathways, and possibly involves a series of humoral factors, such as antimicrobial peptide CEC3, CLIP serine proteases, and lysozymes, making mosquitoes more resistant to pathogen infection [13]. Therefore, 20E can enhance the immune system response and reduce susceptibility to Plasmodium infection. We speculate that after DENV2 infection, Aag2 cells activate the pathway that leads to the expression of the E20MO gene and, subsequently, the activation of certain immune pathways, thereby hindering the replication of DENV2 within the cell. Additionally, the E20MO gene was originally found to be highly expressed in the midgut of larvae of *Drosophila* [8] and most specifically expressed in the midgut of female *Aedes aegypti* mosquitoes [18]. A study has suggested that the determinant of mosquito infection was identified as the DII region of the E protein of DNEV, and changing the DII region significantly enhanced *Aedes aegypti* midgut infection [30]. Therefore, the E20MO gene may influence the recognition or interaction of the host organism with the E protein of DENV2, thereby leading to an increased susceptibility of Aag2 cells to DENV2 infection upon the knockout of the E20MO gene. These hypotheses need to be tested through experiments.

## 5. Conclusions

The present study contributes novel insights into the impact of the E20MO gene on DENV2 replication. Our findings suggest that the observed effects are potentially mediated by the activation of intracellular defense mechanisms and may be associated with a diverse array of soluble factors that bolster mosquito resistance to DENV2 infection. Notably, existing literature has not established a direct correlation between viral infection and the E20MO gene. To our knowledge, we are the first to propose that the deletion of the E20MO gene may promote DENV2 replication in Aag2 cells, shedding light on the potential role of this gene in the Aag2 cell response to mosquito-borne viruses and offering new perspectives on its functional significance. Nonetheless, further investigation is warranted to delineate the precise mechanisms underlying the operation of the E20MO gene in this context.

## Data Availability

All data generated or analyzed during this study are included in this published article.
